# Assessment of Abrasion-Induced Visual Defects in Twin Screw Wet Granulation Using Wall Friction Measurements

**DOI:** 10.1208/s12249-021-02140-5

**Published:** 2022-01-04

**Authors:** Judith Menth, Martin Maus, Karl G. Wagner

**Affiliations:** 1 Pharmaceutical Development, Boehringer Ingelheim Pharma GmbH & Co. KG, Birkendorfer Straße 65, 88397 Biberach, Germany; 2 Department of Pharmaceutical Technology, University of Bonn, Gerhard-Domagk-Straße 3, 53121 Bonn, Germany

**Keywords:** abrasion, twin screw granulation, wall friction, ring shear tester, continuous granulation

## Abstract

Starting point of the presented study were abrasion effects occurring during a twin screw wet granulation (TSG) process of a new chemical entity (NCE) formulation, resulting in gray spots on the final tablets. Several actions and systematic changes of equipment and process parameter settings of TSG process were conducted which reduced the visual defect rate of the tablets, i.e., gray spots on the surface, below the specification limit. To understand the rationale and mechanism behind these improvements, correlations of defect rates and wall friction measurements using a Schulze ring shear tester were evaluated. To check the suitability of the method, a broad range of wall materials as well as powder formulations at various moisture levels were investigated with regard to their wall friction angle. As differences in wall friction angle could be detected, further experiments were conducted using wall material samples made out of different screw materials for TSG. Evaluation of these screw wall material samples gave first hints, which screw materials should be preferred in regard of friction for TSG process. In the finally presented case study, wall friction measurements were performed using the above mentioned NCE formulation with known abrasion issues at TSG processing. The results confirmed that changes which led to a reduced visual defect rate of tablets correlated with a decreased wall friction angle. The results suggest wall friction measurements as a potent tool for equipment selection and establishment of a suitable process window prior to conducting TSG experiments.

**Graphical abstract:**

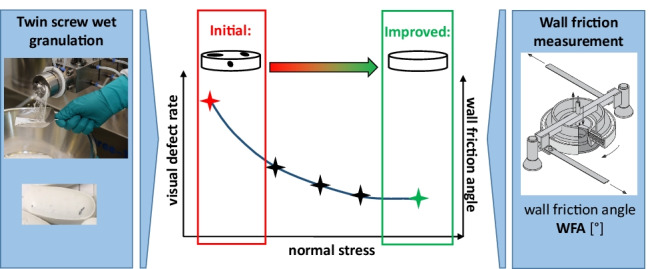

## Introduction

The aim of pharmaceutical production is to always result in a final drug product of the desired quality. Key for this aim is proper process understanding, including potential issues and their associated risks, which may occur during a pharmaceutical process. For twin screw granulation, one important process immanent factor is friction. For good, to facilitate dispersive and distributive mixing [1], but also for bad in case pronounced abrasion of screw and/or barrel material occurs. To understand and balance the effect of friction, a supportive method should facilitate a more rational selection of equipment and process settings. To evaluate friction effects between powders and equipment, wall friction measurements are supposed by many authors [2–13]. The measured parameter describing the friction between a powder and a (equipment) surface is the wall friction angle, which is accessible by various wall friction testers described in literature [2, 6, 12, 14]. Amongst the listed, the Schulze ring shear tester offers easy handling of small sample sizes (appr. 10 g for wall friction measurement) at reduced measuring periods [14] and was therefore selected to be used in this study. Apart from this practical considerations, Schulze described the big advantage of ring-shaped shear cells as they allow to apply a not limited shearing path [15]. One common application wall friction testers are used for is the design of silos. Bradley et al. [4], Jager et al. [11], and Prescott et al. [13] pointed out the importance of the knowledge about wall friction to guarantee mass flow inside a silo or bin.

In addition, several authors have investigated the influence of either powder material or wall material attributes on the wall friction angle. Fekete et al. [5], Halford et al. [7], and Iqbal et Fitzpatrick [10] described the impact of powder moisture on resulting wall friction angle in their work. In general, they all found the effect of increasing wall friction at increasing moisture level of the powder. Additionally, Fekete et al. found a pronounced decrease of that relationship exceeding a certain moisture level [5] and Halford et al. found the described relationship especially at a lower normal pressure [7]. Bradley et al. could show the correlation of increasing relative wear of different steel materials with increasing wall friction angle [3]. Han [8] found lower wall friction angles for polished steel compared to non-polished samples.

The interaction of powder and process dependence on friction, adhesion, and cohesion phenomena are mostly described for the tableting process [16–19].

Nakamura et al. used a shear stress measurement device for predicting the sticking tendency of powder to the punch using friction measurements [18], which might be mechanistically similar to phenomena observed in twin screw granulation.

Inspired by the above described works, the presented work focuses on the twin screw wet granulation process. Twin screw (wet) granulation has been gaining more and more interest within the last years as it offers the possibility of a continuous way to perform wet granulation which result in several advantages, e.g., reduced footprint, higher flexibility, and increase of product quality by diversion of bad fraction material [20]. Focus within this study was set on how abrasion effects potentially occurring during that process can be explained using wall friction measurements. Regarding abrasion process/equipment settings as well as formulation aspects have to be considered to be potential impact factors. The screw configuration is one important equipment setting affecting friction effects inside the twin screw granulator. Simple conveying elements as well as kneading elements are commonly used screw elements for TSG process [21]. Stauffer et al. already considered a temperature increase along the barrel to be proportional to granulator power consumption and friction forces [1] in the zones where kneading elements were applied. Kumar et al. found an increase of torque with the increasing number of kneading elements being added to the screw [22]. That clearly shows that higher friction forces are generated by adding kneading elements to the simple conveying elements. Important formulation aspects are the filler combination as well as the selection of binder. The two binders being used within this study are Copovidone and HPMC. Higher binder evectiveness of Povidone compared to HPMC regarding granulation process was found by different researchers [23, 24]. Nevertheless, Vandevivere et al. did not find a correlation between binder effectiveness and torque within their study [24].

Explanation of friction forces occurring inside the twin screw granulator at distinct equipment and process parameter settings of TSG and for several formulations as well as the correlation of the impact of these parameters on wall friction angle is the aim of this paper. Therefore, wall friction measurements were performed using a Schulze ring shear tester, investigating the impact of various wall/screw materials, powder blends, moisture levels, and normal stress levels. The obtained impacts will be applied to a NCE case study to explain the empirical process optimization in respect of reduced material abrasion and subsequently, limited visual defects on resulting tablets.

## Material and Methods

### Material

#### Formulation 1

Formulation 1 was based on the two fillers Mannitol (MAN, Pearlitol 50 C, Roquette GmbH) and microcrystalline cellulose (MCC, Avicel PH-101, FMC Biopolymer) in a ratio of 2:1 (w/w). For this formulation, a placebo and an active preblend were prepared. For the active formulation, Acetaminophen micronized (APAP, Paracetamol micronized, Mallinckrodt Pharmaceuticals) at a drug load DL = 5% was added as a model active pharmaceutical ingredient (API). The entire amount of the binder Hypromellose (HPMC, Methocel E5 – LV USP/EP Prem., Dow Chemical Company) and half of the amount of the disintegrant Crospovidone (CROS, Kollidon CL-SF, BASF SE) were added to the preblend consisting of APAP (only for the active formulation), MAN, and MCC, to result in the intragranular phase. This preblend was used for the investigation of wall friction angle via the ring shear wall friction method as described later. The detailed composition of formulation 1 including the extragranular phase is described in Table I.

**Table I:** Composition of Formulation 1 (Placebo and Active)

Excipient/API	Placebo: weight percentage [% (w/w)]	Active: weight percentage [% (w/w)]	Function – formulation part
APAP	-	5.0	**API**—intragranular
MAN	63.0	59.7	**filler**—intragranular
MCC	31.5	29.8	**filler**—intragranular
HPMC	1.5	1.5	**binder** – intragranular
CROS	1.5	1.5	**disintegrant**—intragranular
CROS	1.5	1.5	**disintegrant**—extragranular
MGST	1.0	1.0	**lubricant** – extragranular

*Abbreviations: *APAP, Paracetamol micronized; MAN, Mannitol; MCC, Microcrystalline cellulose; HPMC, Hypromellose; CROS, Crospovidone; MGST, Magnesium stearate

#### Formulation 2

Comparable to formulation 1, a placebo as well as an active formulation including APAP at a DL = 5% as model API were prepared for formulation 2. This formulation was based on the two fillers MAN and Starch undried (STA, Maisstärke extra-weiss, Roquette GmbH) in a ratio of 9:1 (w/w). For wall friction measurements, Copovidone (COPV, Kollidon VA 64, BASF SE) as a binder, and CROS, as disintegrant, were added to the intragranular phase. Table II gives an overview of the composition of formulation 2 also including the extragranular phase.

**Table II:** Composition of Formulation 2 (Placebo and Active)

Excipient/API	Placebo: weight percentage [% (w/w)]	Active: weight percentage [% (w/w)]	Function – formulation part
APAP	-	5.0	**API**—intragranular
MAN	82.8	78.3	**filler**—intragranular
STA	9.2	8.7	**filler**—intragranular
COPV	4.0	4.0	**binder** – intragranular
CROS	1.5	1.5	**disintegrant**—intragranular
CROS	1.5	1.5	**disintegrant**—extragranular
MGST	1.0	1.0	**lubricant** – extragranular

*Abbreviations: *APAP, Paracetamol micronized; MAN, Mannitol; STA, Starch undried; COPV, Copovidone; CROS, Crospovidone; MGST, Magnesium stearate

#### BIxx1 formulation

Within the case study, a formulation including the NCE BIxx1 (blinded API, Boehringer Ingelheim Pharma GmbH & Co.KG) at a DL = 20% was evaluated. The formulation consisted mainly of the two fillers MAN and MCC; COPV was added as a binder. Highly dispersed silicon dioxide (SIL; CAB-O-SIL M5P, Cabot Rheinfelden GmbH & Co.KG) improved the flowability and half of the amount of CROS was added to the intragranular phase as disintegrant. A detailed composition is given in Table III.

**Table III:** Composition of the Case Study Formulation Including BIxx1

Excipient/API	Weight percentage [% (w/w)]	Function –formulation part
BIxx1	20.0	**API**—intragranular
MAN	47.0	**filler**—intragranular
MCC	23.5	**filler**—intragranular
COPV	2.5	**binder** – intragranular
SIL	1.0	**glidant—**intragranular
CROS	2.5	**disintegrant**—intragranular
CROS	2.5	**disintegrant**—extragranular
MGST	1.0	**lubricant** – extragranular

*Abbreviations: *APAP, Paracetamol micronized; MAN, Mannitol; MCC, Microcrystalline cellulose; COPV, Copovidone; SIL, Highly dispersed Silicon dioxide; CROS, Crospovidone; MGST, Magnesium stearate

## Methods

### Preparation of Powder Preblends

#### Placebo Preblends – Formulation 1 and Formulation 2

For each of the two placebo formulations (see Tables I and II), a powder preblend of 73.125 kg was prepared. All intragranular excipients were sieved through a 1.016 mm grater holed screen with a Comil 197 S (Quadro Engineering, Canada) at a rotator speed of 1510 rpm. In a second step, the intragranular excipients were blended in a 400 L container freefall blender (Servolift GmbH, Germany) at a rotation speed of 10 rpm for 15 min.

#### Active Preblends – Formulation 1 and Formulation 2

An amount of 97.5 kg of powder preblend was prepared for each of the two active formulations (see Tables I and II). All intragranular excipients except MAN (i.e., MCC, HPMC, CROS for formulation 1; STA, COPV, CROS for formulation 2) were mixed with APAP in a 400-L container freefall blender (Servolift, GmbH, Germany) at a rotation speed of 10 rpm for 5 min. After the blending step, the premix was sieved together with the MAN through a 1.016 mm grater holed screen with a sieving machine. The sieving machine was a Comil 196/2 (Quadro Engineering, Canada) running at a rotator speed of 630 rpm for formulation 1 and a Comil 197S (Quadro Engineering, Canada) running at a rotator speed of 1510 rpm for formulation 2. Finally, all intragranular excipients including the API were blended at a rotation speed of 10 rpm for 10 min in the same 400 L container freefall blender.

#### BIxx1 Formulation

For the case study of the BIxx1 formulation, 8.2 kg of powder preblend were prepared. In a first step, MAN was sieved through a 1.016-mm grater holed screen using a Comil U5 (Quadro Engineering, Canada) at a rotator speed of 1670 rpm. Subsequently, all excipients were blended in a 40 L container freefall blender (Servolift GmbH, Germany) at a rotation speed of 10 rpm and a blending time of 32 min. The resulting excipient premix was sieved through a 1.016 mm grater holed screen using a Comil U5 (Quadro Engineering, Canada) at a rotator speed of 1670 rpm. In the next step, BIxx1 was added to the sieved excipients premix. Finally, the excipients premix plus BIxx1 were blended in a 40-L container freefall blender (Servolift GmbH, Germany) at a rotation speed of 10 rpm for another 32 min resulting in the final powder preblend.

### Wall Friction Measurement with a Ring Shear Tester

#### Method Description – Wall Friction Measurement

**Fig. 1:**
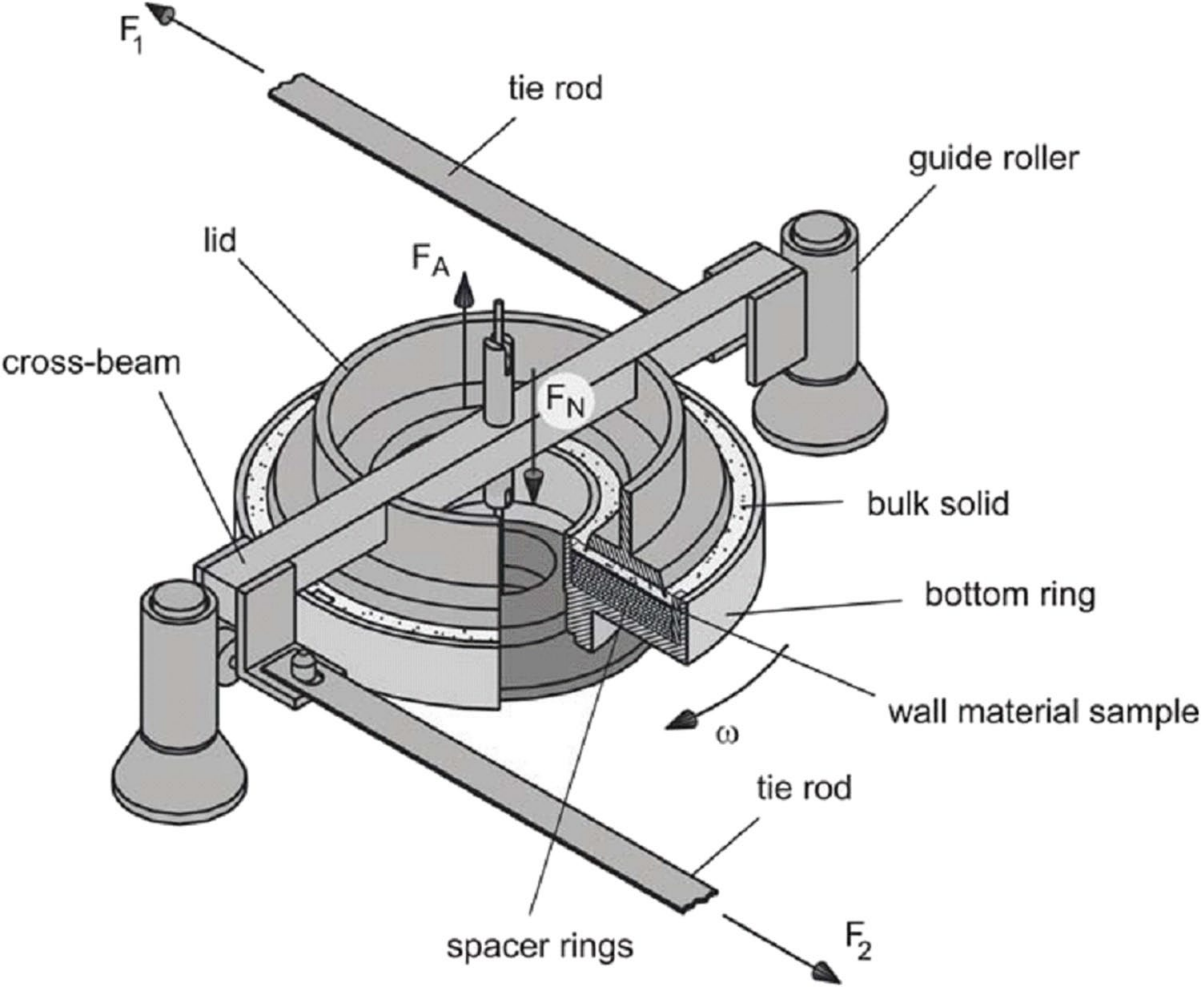
Setup of Schulze ring shear tester for wall friction angle measurement (from Schulze [25])

Wall friction measurement was performed using a Schulze ring shear tester RST-XS.s (Dr.-Ing. Dietmar Schulze Schüttgutmesstechnik, Germany). Measurements were carried out as described by Schulze [15]. A small layer of powder is being sheared across the respective wall material sample, which is placed as insert at the bottom of the shear cell. The shear cell is rotated slowly with a certain angle velocity ω while applying a certain normal stress σ_*w*_ [Pa] on the system (see Fig. 1).

**Fig. 2:**
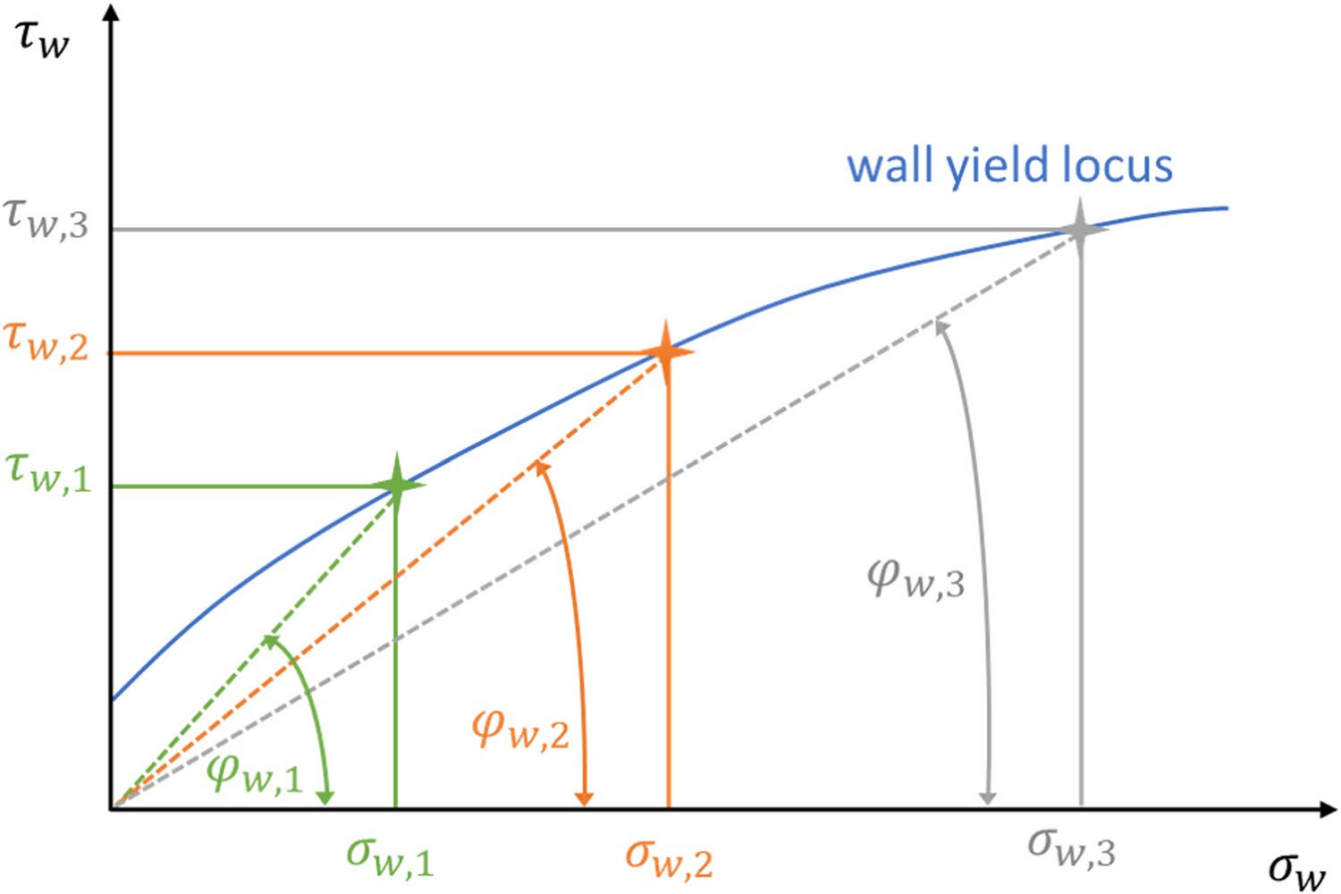
Relationship between shear stresses τ_*w*_ and normal stresses σ_*w*_ resulting in a specific wall yield locus and wall friction angle ( modified from Schulze [25]), reduced wall friction angle at increased normal stress (index 1–3)

Two force sensors record the forces *F*_*1*_ and *F*_*2*_ resulting in the shear stress τ_*w*_ [Pa]. By plotting the shear stress τ_*w,n*_ [Pa] against normal stress σ_*w,n*_ [Pa], a wall yield locus is obtained (see Fig. 2); finally, the wall friction angle φ_*w,n*_ (WFA) [°] per normal stress σ_*w,n*_ [Pa] is calculated according to Eq. (1) described by Behres et al. [14].

1 $$WFA {{\varphi }}_{w,n}={arctan}(\frac{{\tau }_{w,n}}{{\sigma }_{w,n}})$$

Hancock mentioned the typical correlation of normal stress σ_*w*_ [Pa] and WFA φ_*w*_ [°] for pharmaceutical bulks where WFA increases at decreasing normal stress (see also Fig. 2) [9].

#### Method Description – Testing Procedure

For the wall friction measurements presented in this paper, eight normal stresses σ_*w*_ [Pa] were applied in a range of 200 Pa and 4000 Pa. Measurement points were exponentially distributed: σ_*w*_ 1 = 4000 Pa, σ_*w*_ 2 = 2396 Pa, σ_*w*_ 3 = 1453 Pa, σ_*w*_ 4 = 898 Pa, σ_*w*_ 5 = 571 Pa, σ_*w*_ 6 = 379 Pa, σ_*w*_ 7 = 266 Pa, σ_*w*_ 8 = 200 Pa. Within one measurement, this cycle of eight different normal stresses was repeated four times. The average WFA φ [°] in the presented results is the average value of these four repetitions per normal stress σ_*w*_. WFA were measured for the different formulations and their interaction with different wall/screw materials. Furthermore, in addition to the established method to evaluate dry powders and their friction effects on different wall and screw materials, also wet granules (wetted powder) were evaluated comparably. The preparation of the wet granule mass for the wall friction measurement is described later.

#### Wall/Screw Materials

Two sets (named “set 1” or “set 2” in the following) of wall material samples were used for the evaluation in the ring shear tester with regard to their interaction with the formulations described previous.

Three different materials with specific surface treatments were chosen to be included into set 1. Intention for the selection of set 1 materials was to evaluate whether the described wall friction method can differentiate between presumably highly different wall materials and the resulting wall friction of different formulations and moisture levels. Surface roughness of all wall materials (set 1 and set 2) was measured using a MahrSurf M 300 (Mahr GmbH, Germany). The measured roughness Ra was chosen as reference parameter for the surface roughness of each wall material.

Table IV gives an overview of the wall material samples of set 1 = proof of feasibility set.

**Table IV:** Wall Material Samples Placed on the Bottom of the Shear Cell of Set 1 (General Material Screening) with Material Specification, Surface Treatment, and Surface Roughness Ra [µm]

Sample no	Material specification	Surface treatment	Surface roughness (µm)
1	Glass	Smooth (untreated)	Ra: 0.006
2		Rough	Ra: 2.299
3	Plastic: PEEK	Smooth	Ra: 3.548
4	Steel type 1.4301	Cold-rolled	Ra: 0.171
5		Polished	Ra: 0.163

*Abbreviations: *PEEK, Polyetheretherketone

**Fig. 3:**
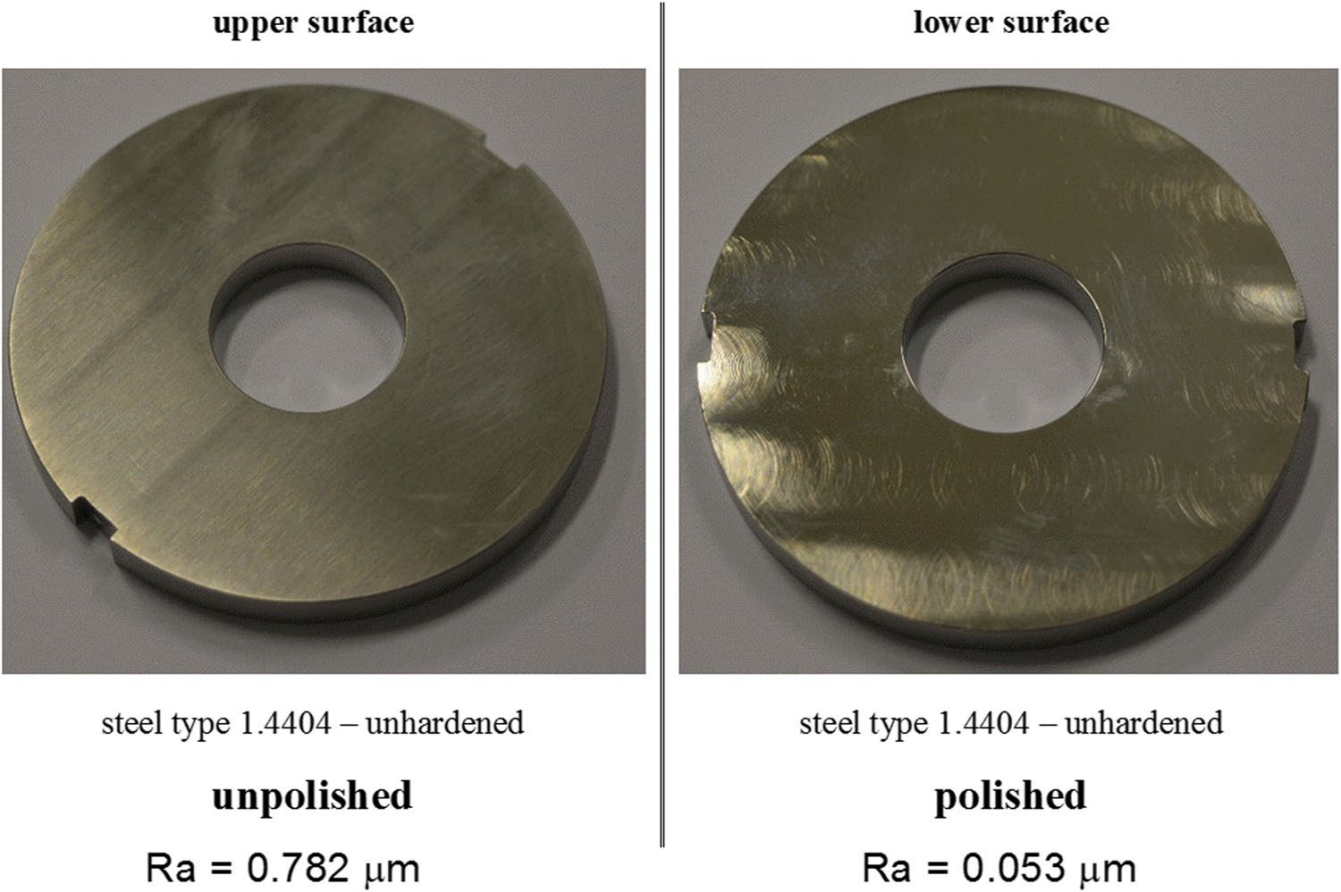
Visual difference between the unpolished and polished surface of wall material testing inserts for one exemplary screw material sample (1.4404 unhardened); positioned on bottom of shear cell

In contrast to set 1, wall material sample set 2 consisted of more similar, but still different screw materials being under current evaluation for twin screw wet granulation processes within the NCE development unit of Boehringer Ingelheim Pharma GmbH & Co. KG. Five wall material samples had therefore been manufactured by Three Tec GmbH, Switzerland. These five samples were made of the two steel types (1.4404 or 1.4542). Both steel types were either used untreated or surface hardened by the described means (see Table V). Each of the five samples had an unpolished side and a polished side (exemplary shown for one sample in Fig. 3).

**Table V:** Wall/Screw Material Sample Description of Set 2 (Specific Screw Materials) with Material Specification, Special Surface or Hardening Treatment, and the Surface Roughness Ra [µm]

Sample no	Material specification	Surface/hardening treatment	Roughness of final surface (µm)
1	Steel type 1.4404	Unhardened	Polished – Ra 0.053
			Unpolished – Ra 0.782
2		HARD-INOX®-S hardened	Polished – Ra 0.091
			Unpolished – Ra 0.531
3	Steel type 1.4542	Unhardened	Polished – Ra 0.531
			Unpolished – Ra 0.570
4		Precipitation hardened	Polished – Ra 0.053
			Unpolished – Ra 0.610
5		TiN layered	Polished – Ra 0.283
			Unpolished – Ra 0.812

*Abbreviations: TiN*, titanium nitride

Table V shows the complete list of wall/screw material samples for set 2 = evaluation set.

#### Preparation of Wet Granule Mass for Wall Friction Measurement

Wetting of dry powder material to result in a wet granule mass was performed by gentle blending and kneading using mortar and pestle. According to Eq. (2), the amount of needed water to be added to the dry powder preblend to result in a certain granulation moisture level ML [%] was calculated. For the different formulations (see Tables I, II and III), different MLs were chosen as the evaluated formulations require different granulation MLs for successful granulation. For formulation 1 (see Table I), the MLs were ML1 = 0% (dry powder preblend), ML2 = 20%, ML3 = 30%, and ML4 = 40%.

For formulation 2 (see Table II), the MsL were ML1 = 0% (dry powder preblend), ML2 = 10%, ML3 = 15%, and ML4 = 20%.

For the BIxx1 formulation (see Table III), the investigated MLs were ML1 = 25% and ML2 = 30%.

2 $${mass}_{water}\left[g\right]=\frac{\frac{ML [\%]}{100} * {mass}_{powder}[g]}{1-\frac{ML [\%]}{100}}$$

### Twin Screw Wet Granulation Process

Twin screw wet granulation experiments were performed using the BIxx1 formulation (see Table III). A ZE 16 granulator (Three-Tec GmbH, Switzerland) with a diameter of screws D = 16 mm was used as twin screw granulator. The L/D-ratio (length-to-diameter-ratio) of the granulator was 32. Screws were equipped with conveying elements only. For continuous feeding of the powder preblend into the granulator, a ZD12 flat bottom volumetric feeder (Three-Tec GmbH, Switzerland) was applied. A peristaltic tube pump (Watson Marlow GmbH, Germany) was used for adding water as granulation liquid into the granulator.

The power consumption [A] was recorded during processing of at least several process parameter settings and enabled to calculate granulator torque for these settings. The specific mechanical energy was calculated according to Bochmann et al. [26].

### Visual Defect Rate of Tablets

**Fig. 4:**
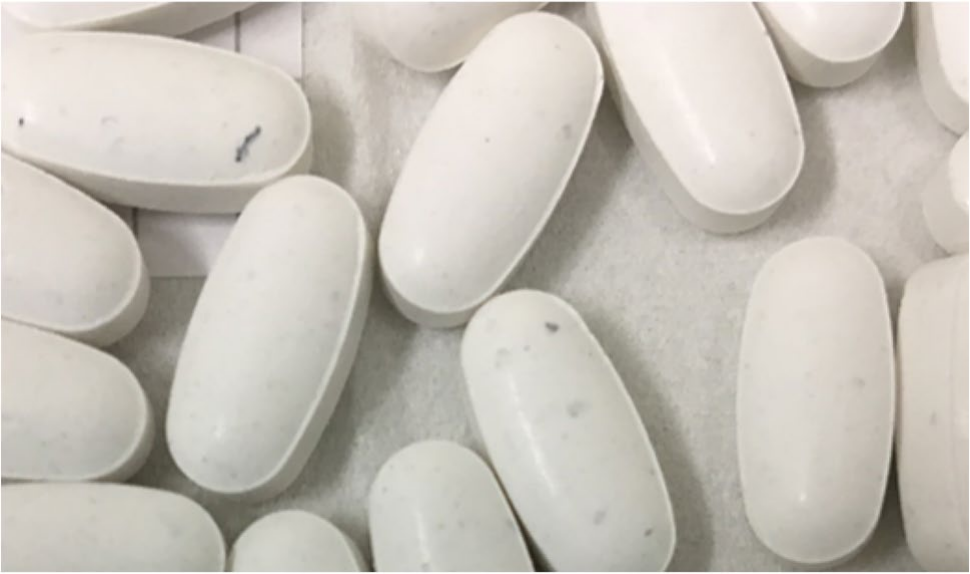
Tablets with visual defects caused by abrasion effects during twin screw wet granulation process

Visual defect rate of tablets was determined after tableting for a defined sample size (*n* = 200) which was appr. 6% of the total batch size in that case. The tablet sample was visually inspected from both sides and all tablets with slight spots as well as specks were sorted out. An example of how tablets with spots and specks looked like is given in Fig. 4.

## Results

### Wall Friction Measurement Method – Proof of Feasibility

**Fig. 5:**
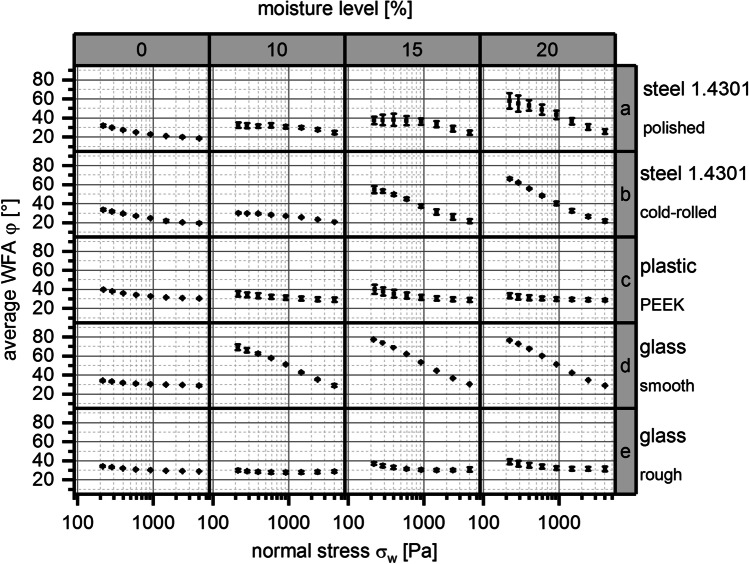
Relationship between average wall friction angle WFA φ [°] and normal stress σ_*w*_ [Pa] for placebo formulation 2; error bars, ± 1 SD (*n* = 4); rows, wall materials; columns, moisture level [%]

Figure 5 shows the interaction of placebo formulation 2 with the different wall materials of set 1 (see Table IV) at different granulation moisture levels [% (w/w)] and normal stresses σ_w_ [Pa].

A decrease of the average WFA φ [°] with increasing normal stress *σ*_*w*_ [Pa] was observed. Furthermore, an increase of average WFA φ [°] with increasing moisture level [%] - compared at defined normal stresses *σ*_*w*_ [Pa] - was obtained. These two effects were especially pronounced for steel with the two different surface treatments (polished and cold-rolled) and glass with a smooth surface. The plastic wall material PEEK (polyetheretherketone) and the glass material with roughened surface did not show a comparable strong effect of normal stresses *σ*_*w*_ [Pa] and moisture level [%] on average WFA φ [°]. Additionally, differences in average WFA φ [°] were observed between the different wall materials and surface treatments at fixed normal stress *σ*_*w*_ [Pa] and comparable moisture level [%]. For example, at a normal stress *σ*_*w*_ = 200 Pa and a moisture level ML = 15%, the lowest WFAs (φ = 37° to 40°) were measured for the wall materials glass with roughened surface, the plastic PEEK, and the polished surface of the 1.4301 steel. A medium WFA (φ = 55°) was measured for the cold-rolled surface of the 1.4301 steel. The highest WFA (φ = 77°) was obtained for the glass material with a smooth surface.

**Fig. 6:**
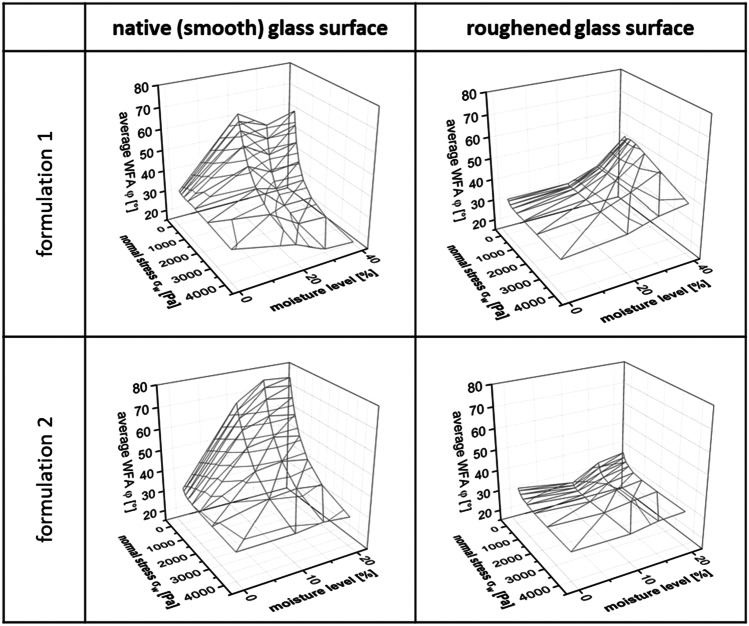
Relationship between average wall friction angle WFA φ [°], normal stress σ_w_ [Pa], and different moisture levels [%]; columns, glass surfaces; rows, placebo formulations 1 and 2

Using an exemplary wall material (glass – roughened and smooth surface), Fig. 6 illustrates how differences in formulations affected the measured WFA φ [°].

Looking at the results of the average WFA φ [°] at different moisture levels [%] and normal stresses *σ*_*w*_ [Pa] on smooth glass surface and roughened glass surface for both placebo formulations shows that average WFAs φ [°] were lower on the roughened surface compared to the smooth surface irrespective of the investigated formulation. Furthermore, average WFAs φ [°] were very similar for both formulations with regard to friction on the roughened glass surface. In contrast to that, pronounced differences between formulation 1 and formulation 2 were visible for the experiments performed on the smooth surface. Starting at ML = 0%, formulation 1 showed a maximum of WFA at a ML = 20%; at higher ML, lower WFA values were observed again. In contrast to that, formulation 2 showed a strong correlation of WFA φ [°] and normal stress *σ*_*w*_ [Pa], and moisture level [%]. WFA φ [°] steadily increased at decreasing normal stress *σ*_*w*_ [Pa] and increasing moisture level [%].

### Evaluation of Wall Friction Angle for Different Screw Materials and Formulations

**Fig. 7:**
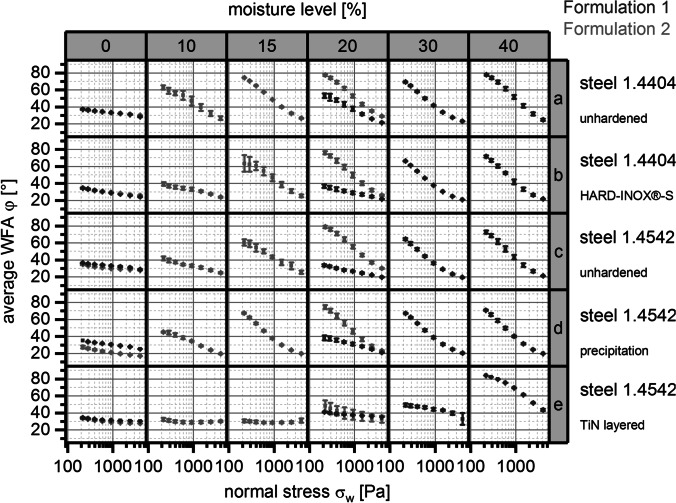
Relationship between average wall friction angle WFA φ [°] and normal stress σ_*w*_ [Pa] for active formulation 1 and 2; error bars, ± 1 SD (*n* = 4); columns, moisture levels [%]; rows, screw materials with polished surface

In a second step, experiments with samples made of different screw materials were conducted to check if the results from the method described in the previous chapter can be linked to the twin screw wet granulation process. The description of the wall/screw material set 2 can be found in Table V. The correlation of average WFA φ [°] and normal stress *σ*_*w*_ [Pa] for both active formulations 1 and 2 (see Tables I and II), different moisture levels [%], and all investigated screw materials of set 2 with polished surfaces is depicted in Fig. 7.

Except for formulation 2 in the interaction with TiN (titanium nitride)-layered type 1.4542 steel surface, the interactions between all other screw materials and both formulations resulted in decreasing average WFA φ [°] with increasing normal stress σ_w_ [Pa] and decreasing moisture level [%]. These correlations were also described for wall material set 1.

**Fig. 8:**
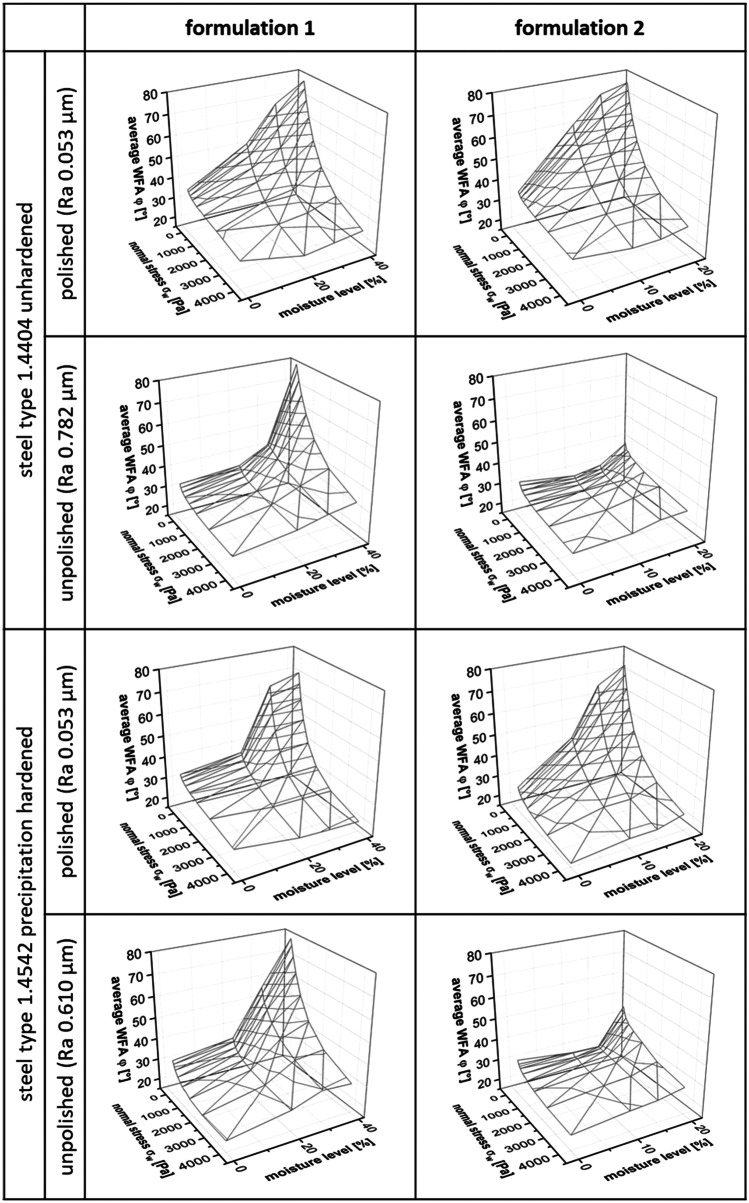
Average wall friction angle WFA φ [°] in relation to different normal stresses σ_w_ [Pa] and moisture levels [%]; rows, screw materials and surface treatments; columns, active formulations 1 and 2

Nevertheless, differences of average WFA φ [°] could be detected as illustrated in Fig. 8.

WFAs φ [°] did differ between formulations, screw materials, and surface treatments of these screw materials (polished *vs.* unpolished), as well as on moisture level [%] and normal stress *σ*_*w*_ [Pa]. Two screw materials - steel type 1.4404 unhardened and steel type 1.4542 precipitation hardened - were exemplarily chosen for further evaluation of the TSG process.

Considering the surface roughness Ra of polished and unpolished materials, it was found that for almost all materials the values of Ra of polished surfaces are 10 times smaller compared to the unpolished. Different effects of the surface roughness on the WFAs of the evaluated two formulations were found. While formulation 1 did not show big differences between the two surface treatments, formulation 2 showed the “typical” correlations as described earlier between WFA and normal stress regarding moisture level for polished surfaces of screw material, but not for unpolished surfaces. Interestingly, the WFAs describing the interaction between formulation 2 and unpolished screw surfaces were comparatively low (φ < 50°), showing a completely different 3D plot (see Fig. 8) when compared to their “polished” partner-experiment.

Comparing two specific screw materials with polished surfaces - type 1.4404 unhardened steel and type 1.4542 precipitation hardened steel - average WFA was lower for the type 1.4542 precipitation hardened steel screw than for the type 1.4404 unhardened steel screw. This observation was found for both formulations.

### Case Study

#### Abrasion Effects During TSG Process

As described by Menth et al. [27], gray spots occurred on the surface of tablets made of TSG granules using the BIxx1 formulation as described previous. These spots resulted from gray discoloration of the powder accumulating as a film at the barrel wall during the TSG process and were suspected to result from abrasion effects. The resulting ratio of tablets showing visual defects was 46.5% related to all tested tablets, at the maximum (representative picture of tablets with visual defects, see Fig. 4). Several actions were taken to reach a ratio of being affected to be visually rated tablets below the specification limit of 6.5% of tablets. Table VI gives an overview on these actions.

**Table VI:** Actions Taken to Improve TSG Process with Regard to Process Parameter Settings and Equipment Settings with the Aim to Minimize Visual Defect Rate of Resulting Tablets

		Initial: visual defect rate of tablets **above** specification limit	Improved: visual defect rate of tablets **below** specification limit
Process parameter setting TSG process	Powder feed rate PFR [kg/h]	0.9	2.6
	Liquid feed rate LFR [kg/h]	0.3	1.1
	Screw speed granulator [rpm]	150	329
	Granulation moisture level ML [%]	25	30
	Filling level FL inside barrel [%]	6.5	8.6
Equipment settings TSG process	Granulator screws	steel type: 1.4404 unhardened; modular screw elements	steel type: 1.4542 precipitation hardened; monolithic screw
	Cooling of barrel	No cooling	Water cooling

Process parameters were adapted to result in higher shear forces by increasing the powder feed rate PFR [kg/h] and screw speed [rpm]; this consequently resulted also in a higher filling level FL [%]. Furthermore, granulation moisture level ML [%] was increased. Granulator screws were exchanged as given in Table VI. An additional water cooling system was installed to the granulator barrel to keep the barrel temperature at a maximum of appr. 28 °C even for process runs exceeding 15 min.

The described optimizations to affect the smooth transport of wetted material do reflect the before mentioned reduction of WFAs and surprisingly did show a positive effect in the production process.

In summary, the sum of the actions was successful, resulting in a visual defect rate of the tested tablets within specification limit of lower than 6.5%.

#### Supportive Measurements Understanding TSG Process

##### Evaluation of Granulator Torque

**Fig. 9:**
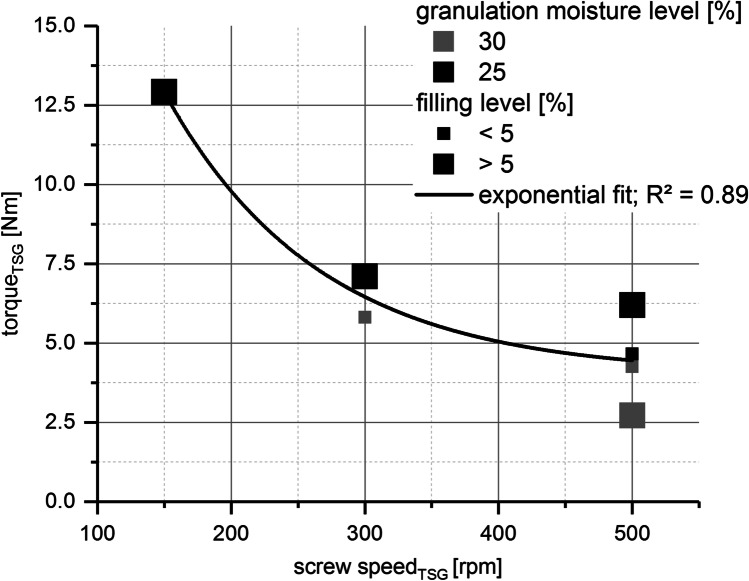
Influence of granulator screw speed [rpm] on granulator torque [Nm] using BIxx1 formulation; colors, granulation moisture levels [%]; bullet point size, barrel fill levels FL [%]

Conductive power consumption measurements [A] of the twin screw granulator motor enabled granulator torque output [Nm] for several different process parameter settings. As presented in Fig. 9, the major impact on granulator torque is given by granulator screw speed. At a low screw speed of 150 rpm, the granulator torque reached its highest value (12.9 Nm). For a middle screw speed of 300 rpm, a granulator torque range between 5.8 and 7.1 Nm was reached. At the highest investigated screw speed of 500 rpm, the granulator torque decreased further to a range between 2.7 and 6.2 Nm.

This interesting observation led to the authors’ expectation that wall friction measurements with a ring shear tester showing similar trends (decreasing abrasion at increasing normal stress) could serve as an additional supportive method addressing the explanation of abrasion effects inside TSG. The finding of decreasing torque at increasing screw speed was also investigated by several other research papers [22, 28, 29].The influences of barrel filling level FL [%] (affected by the changes in throughput) and granulation moisture level ML [%] on granulator torque were only minor within this study.

##### Evaluation of WFA Measurements Using a Ring Shear Tester

**Fig. 10:**
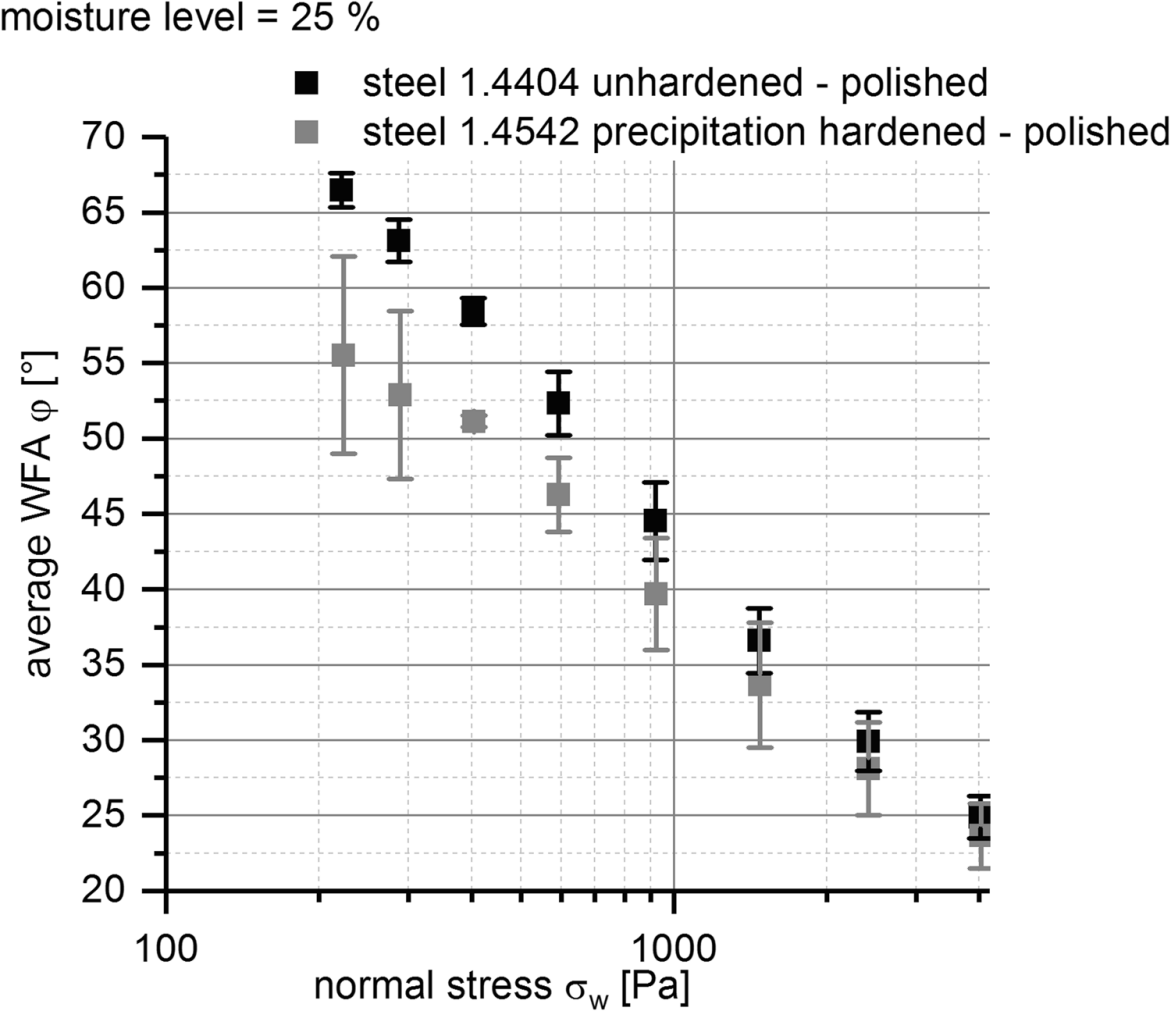
Average wall friction angle WFA φ [°] *vs.* normal stress σ_w_ [Pa] at given granulation moisture level for BIxx1 formulation; error bars, ± 1 SD (*n* = 4); colors, screw materials

As already described previous, in addition to the evaluation of granulator torque [Nm], wall friction measurements with the BIxx1 formulation were conducted. The purpose of that measurements was to evaluate whether they may help to explain why the actions described in Table VI led to the improvement in terms of abrasion effects during TSG process. Figure 10 shows the results of wall friction measurements performed with a wet granule mass of the case study formulation at a ML = 25%. Average WFAs are plotted against normal stress for the two different screw materials used for the TSG process.

As expected, the average WFAs decreased for both screw materials with increasing normal stress. Comparing the screw materials, average WFAs for the precipitation hardened type 1.4542 steel screw were lower than for the unhardened type 1.4404 steel screw for all applied normal stresses.

In a second step, the impact of different granulation moisture level ML on average WFA was investigated. The interaction between the BIxx1 formulation and the precipitation hardened type 1.4542 steel screw was measured at the two moisture levels ML1 = 25% and ML2 = 30%.

**Fig. 11:**
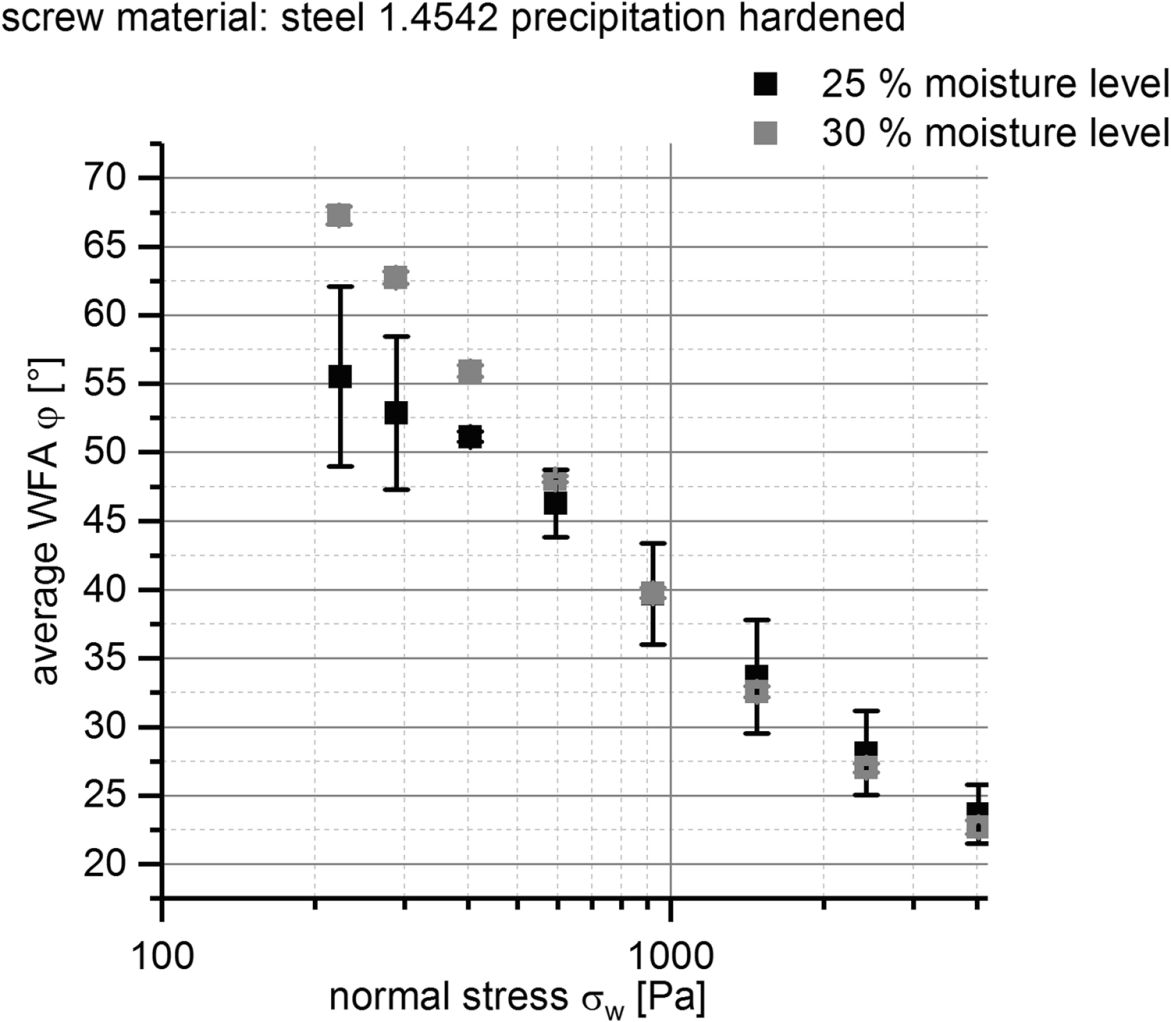
Average wall friction angle WFA φ [°] *vs.* normal stress σ_w_ [Pa] at given screw material for BIxx1 formulation; error bars, ± 1 SD (*n* = 4); colors, moisture levels (%)

As depicted in Fig. 11 for normal stresses, σ_*w*_ 4 < 898 Pa WFAs are lower at ML1 = 25% than at ML2 = 30%. At normal stresses σ_*w*_ 4 > 898 Pa, this relationship switches so that WFAs are a lower at ML2 = 30% than at ML1 = 25% but without showing any significant effects as standard deviations are overlapping for this second case.

## Discussion

The wall friction measurements with wall material set 1 (see Table IV), which offers a high variety of different wall materials in terms of surface roughness and treatment, showed the typical correlations between WFAs and normal stress as also described in literature [9], confirming the usability of wall friction measurements in this regard. Typically, WFA increases at decreasing normal stress, see also Fig. 5 for wall materials steel and smooth glass. Furthermore, granulation moisture level ML were identified to be of high impact on resulting WFAs as well, with WFAs typically increasing with increasing ML (see also Fig. 5). But within this investigation, some exceptions to these two described correlations were observed. For example, the WFAs measured on the wall material plastic PEEK and the glass material with roughened surface were shown to be quite independent of normal stress (see Fig. 5). Another exception occurred. Formulation 1 resulted in a maximum WFAs on the glass material with smooth surface at a ML = 20%, as depicted in Fig. 6. For increasing normal stress at MLs > 20%, the WFAs was either further decreasing or remaining on an equal level when compared to a ML = 20%. The authors expect the “sponge” effect of cellulose [30] being part of formulation 1 (31.5% (w/w)) to be responsible for the described effect, as uptaken water might be released under pressure and shear conditions. This leads to a decrease of wall friction angle dependent on moisture level ML and at increased normal stress.

In the presented work, the WFA method was evaluated regarding its ability to differentiate between different wall materials, surface treatments of wall materials, and formulations.

As wall materials from set 1 were successfully differentiated by the method, experiments were conducted to test screw material set 2 (see Table V). In contrast to wall material set 1, screw material set 2 was much more similar in terms of surface roughness, and treatment. Nevertheless, the impact of normal stress, ML, screw materials including their surface treatment, and formulations on WFA could be measured and differentiated with screw material set 2 as well (see Fig. 7). The TiN-layered screw material showed potential advantages in terms of lower WFAs independent of normal stress in most instances. The evaluation of the two screw materials unhardened 1.4404 steel *vs.* precipitation hardened 1.4542 steel showed the advantage of precipitation hardened 1.4542 steel screw in terms of friction.

Finally, the case study presented enabled to figure out the connections between all evaluated modules:

- abrasion effects occurring inside TSG resulting in a high visual defect rate of tablets
- adaption of process parameter and equipment settings to minimize these abrasion effects
- granulator torque
- wall friction angle

**Fig. 12:**
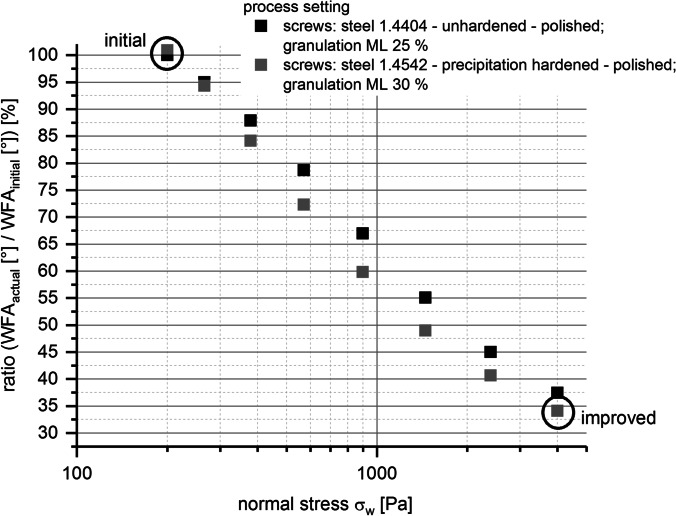
Decrease of measured wall friction angle WFA [°] with a ring shear tester using improved process settings; colors, black – initial settings; gray - improved settings

A clarification of these connections is depicted in Fig. 12 and Table VII. Calculation of specific mechanical energy for the granulator (SME) [kWh/t] offered the possibility to evaluate the influence of granulator screw speed, adaption of powder feed rate, and granulator torque at a specific screw speed (based on the relationship found in Fig. 9) on the improvement of tablets’ visual defect rate.

**Table VII:** Relation Between Specific Mechanical Energy (SME) [kWh/t] (TSG), Measured Wall Friction Angles (WFA) [°] (Ring Shear Tester), and Resulting Defect Rate [%] (Tablets) for Initial and Improved Process Settings

Initial		Improved
225	**TSG** Specific mechanical energy SME [kWh/t]	78
66	**Ring shear tester** Wall friction angle WFA [°]	23
46.5	**Tablets** Visual defect rate_tablets_ [%]	< 6.5

Comparing SME value for initial and improved process settings, a decrease of SME to 34.8% for the improved process settings (SME = 78 kWh/t) compared to the initial process setting (SME = 225 kWh/t) was calculated. The same trend could be figured out within the wall friction measurement. Within the applied range of normal stresses (200–4000 Pa), a decrease of the wall friction angle to 34.1% of WFAs (23°) at highest normal stress of 4000 Pa compared to the WFAs (66°) obtained at lowest applied normal stress (200 Pa) could be found (see Fig. 12 and Table VII), too.

Furthermore, with the help of WFA measurements, additional information with regard to exchange of screw material and adaption of granulation moisture level ML could be evaluated. The advantage of the type 1.4542 steel screws with precipitation hardened surface compared to the type 1.4404 unhardened steel screws in terms of friction and improvement of tablets’ visual defect rate could be shown. WFAs were lower for measurements using the improved screw material compared to measurements conducted with the initial screw material within the whole applied normal stress range (see Fig. 10). The evaluation of the impact of granulation moisture level (ML) on WFAs showed that increasing ML led to either a decrease or increase of WFAs and was dependent on applied normal stress (see Fig. 11). Therefore, the impact of ML on improvement of tablets’ visual defect rate seems to be minor compared to the adaption of the other process parameter settings and the exchange of the screw pair.

All in all, the case study showed that WFA measurements could serve as a base for explanation and justification of the several actions conducted to improve tablets’ visual defect rate (see Table VI) as the correlation to the TSG process could be shown evaluating granulator torque and calculating SME. Adapting process parameter settings by increasing screw speed, throughput and fill level of the granulator as well as adapting equipment settings by exchanging screw material were found to have a high impact on improving abrasion effects inside TSG. Therefore, WFA measurements were shown to be a helpful tool for the assessment of abrasion induced effects inside TSG - either for troubleshooting purposes or already at the start of any experiments.

## Conclusion

An extensive evaluation of WFA measurements using the Schulze ring shear tester for different wall and TSG screw material samples and three different formulations was performed. In addition, the impact of normal stress *σ*_*w*_ as well as granulation moisture level ML on WFAs was investigated for the combination of different wall/screw material samples and different formulations. As expected, WFAs was found to differ depending on combinations of wall/screw material and formulation.

WFA measurements with the different screw materials showed that precipitation-hardened 1.4542 steel screws and TiN-layered 1.4542 steel screws outperformed other screw materials in terms of low WFAs. Therefore, these two screw materials should be preferred for use in TSG process to minimize abrasion effects. In retrospect, WFA measurements helped to understand the successful trouble shooting actions minimizing abrasion effects in a specific case study. WFA measurements gave mechanistic explanations and confirmed the process parameter and equipment changes to the TSG process.

For the future, this application of WFA measurement for TSG screw materials can serve as an easy tool for all stages of pharmaceutical development of new chemical entities as only low amount of drug substance is needed. Based on these measurements, a reasonable decision on TSG equipment material as well as process parameter settings should be possible already at the start of drug product development increasing process knowledge at reduced process risks, i.e., improving quality by design.
